# High casein concentration induces diarrhea through mTOR signal pathway inhibition in post-weaning piglets

**DOI:** 10.3389/fmicb.2024.1430511

**Published:** 2024-09-04

**Authors:** Jing Gao, Li Ma, Yulong Yin, Yongzhong Chen, Tiejun Li

**Affiliations:** ^1^Research Institute of Oil Tea Camellia, Hunan Academy of Forestry, Changsha, China; ^2^National Engineering Research Center for Oil Tea Camellia, Changsha, China; ^3^Key Laboratory of Agro-ecological Processes in Subtropical Region, Institute of Subtropical Agriculture, Chinese Academy of Sciences, Beijing, China; ^4^University of Chinese Academy of Sciences, Beijing, China; ^5^College of Life Science, Hunan Normal University, Changsha, Hunan, China

**Keywords:** dietary protein concentration, weaned piglets, post-weaning diarrhea, solute carrier, gut microbiota, mTOR signaling pathway

## Abstract

Weaning is one of the most challenging periods in a pig’s life, during which piglets suffer from nutrition and other issues. Post-weaning diarrhea is one of the major health problems in the pig industry, leading to high morbidity and mortality rates. Previous studies have demonstrated that both the source and concentration of proteins are closely associated with post-weaning diarrhea in piglets. This study was conducted to prevent and control post-weaning diarrhea by selecting different dietary protein concentrations. To eliminate interference from other protein sources, casein was used as the only protein source in this study. Fourteen piglets (weighing 8.43 ± 0.3 kg, weaned on the 28th day) were randomly assigned to two dietary protein groups: a low-protein group (LP, containing 17% casein) and a high-protein group (HP, containing 30% casein). The experiment lasted 2 weeks, during which all piglets had *ad libitum* access to food and water. Diarrhea was scored on a scale from 1 to 3 (where 1 indicates normal stools and 3 indicates watery diarrhea), and growth performance measurements were recorded daily. The results showed that the piglets in the HP group had persistent diarrhea during the whole study, whereas no diarrhea was observed among piglets in the control group. The body weights and feed intake were significantly lower in piglets in the HP group compared to those in the LP group (*p* < 0.05). The gastrointestinal pH was significantly higher in piglets in the HP group than those in the LP group (*p* < 0.05). The intestinal tract microorganisms of the piglets in both groups were significantly affected by the protein concentration of the diet. A diet with high casein concentration significantly reduced the microbiota diversity. Compared to the LP group, the 30% casein diet decreased the abundance of *Firmicutes*, *Bacteroidetes*, and *Actinobacteria* at the phylum level and the relative abundance of *Ruminococcus* at the genus level. Diarrhea-related mRNA abundances were analyzed by the real-time polymerase chain reaction (PCR) in the intestine of piglets, and the results showed that the HP concentration markedly decreased the expression of solute carriers (SLC, *p* < 0.05). The mammalian target of rapamycin-mTOR signaling pathway (*p* < 0.01) was activated in the HP group. In conclusion, a high-protein diet induced post-weaning diarrhea, decreased growth performance, increased gastrointestinal pH, and reduced expression of solute carrier proteins. However, the relationship between high dietary casein feed and post-weaning diarrhea remains unclear and needs to be explored further.

## 1 Introduction

Piglets suffer from many intestinal diseases after weaning, including irritable bowel syndrome, mucosal inflammation, and post-weaning diarrhea (PWD; Wu et al., 2015). PWD is a multifactorial intestinal disorder, with dietary composition and food sources being the major causative factors (Gao et al., 2019). Previous studies have demonstrated the negative effects of a high-protein diet on weaned piglets (Wu et al., 2015). A high concentration of dietary protein and amino acids is closely associated with the incomplete digestion of excess protein. While some dietary proteins are digested by proteases in the stomach and ileum (such as pepsins, trypsin, and chymotrypsin), the majority of the protein is digested in the colon or cecum by intestinal bacteria (Davila et al., 2013). Undigested proteins and substrates may flow into the large gut from the small intestine, facilitating the proliferation of pathogenic bacteria in the gastrointestinal tract. The fermentation of undigested protein by intestinal microbiota can produce volatile fatty acids and many potentially detrimental metabolites, such as amines and ammonia, which can cause diarrhea (Wu, 2016). However, the presence of undigested protein in the large intestine has been found to be closely associated with pH levels in different sections of the gastrointestinal tract (Fan et al., 2015).

A low stomach pH (2.0–3.5) and a lower intestinal pH (5.0–6.0) are negatively associated with the production of amines and ammonia (Jahan-Mihan et al., 2011). Conversely, a high pH (outside the normal ranges) can stimulate the fermentation of some pathogens in the gastrointestinal tract, which increases the production of amines, ammonia, and other toxic metabolites, and can cause intestinal disorders, including diarrhea (Mall et al., 2002). Importantly, the pH range of the gastrointestinal tract also modulates protease activity (Boyd et al., 2019). The inhibition of protease activity can further inhibit the degradation of excess dietary proteins. In addition, the intestine is the primary organ responsible for sodium (Na^+^) and fluid absorption (Bibbo et al., 2016). Several members of the ion exchange channel protein families, including Na^+^/H^+^ exchangers and Cl^−^/HCO3^−^ exchangers, are expressed in different sections of the gut (Jeffery and O'Toole, 2013). These ion channels contribute to the regulation of Na^+^, H^+^, Cl^−^, and HCO_3_^−^ absorption, intracellular pH balance, and nutrient absorption. Moreover, these ion channels are closely associated with cellular proliferation and apoptosis (Duncan et al., 2009). The disordered expression of ion exchangers is involved in many intestinal diseases, such as diarrhea and inflammatory bowel disease (Vince et al., 1973). Furthermore, protein metabolism can affect the expression of ion exchangers (Wolpert et al., 1970). However, the mechanism of how protein metabolism regulates these changes in pH and ion exchanger expression remains unclear. The piglet model in this study provides a new perspective on the relationship between dietary protein intake and post-weaning diarrhea, and the involvement of the mammalian target of rapamycin-mTOR signaling pathway.

## 2 Materials and methods

### 2.1 Animals and experimental design

The experiment in this study was conducted in compliance with the Chinese guidelines for animal welfare. The experimental protocol was approved by the Animal Care and Use Committee of the Chinese Academy of Sciences; the ethical approval code is ISA2017030523.

A total of 14 piglets (Duroc, Landrace, and Large White; weighing 8.43 ± 0.3 kg) weaned on the 28th day were randomly divided into two treatment groups comprising seven replicates. The piglets were kept in separate metabolism cages.

According to the National Research Council (NRC) 2012 guidelines, low-protein (LP, containing 17% casein) and high-protein (HP, containing 30% casein) diets were prepared and fed to the animals three times per day. The diets satisfied the requirements of all essential amino acids or exceeded the standard of NRC, without adding antibiotics or growth promoters (Table 1).

**Table 1:** Composition of basal diet.

Diets		
Ingredients	17% casein	30% casein
Casein	19.11	33.72
Corn starch	63.39	45.44
Soybean oil	2	0
Sucrose	5	5
Bran	5	5
Milestone power	2	2
Salt	0.5	0.5
Calcium bicarbonate	2	2
Sepiolite	0	5.34
Vitamin-mineral premix	1	1
**Nutrient level**		
Crude protein	16.998	29.994
L-Lysine	1.313	2.317
L-(Methionine +cysteine)	0.568	1.001
L-Threonine	0.72	1.271
L-Tryptophan	0.254	0.448
Total energy	15.305	15.387

^∗^The premix contained the following per kilogram of the diet: sepiolite, 6.043 g; FeSO_4_· + H_2_O, 516 mg; pig vitamin, 750 mg; MnSO_4_· + H_2_O, 250 mg; CoO, 500 mg; ZnSO_4_· + H_2_O, 212 mg; CuSO_4_· + 5H_2_0, 600 mg; Na_2_SeSO_3_, 30 mg; ZnO, 25mg; VB4, 1,000 mg.

The experiment lasted 2 weeks, with the piglets housed individually in temperature-controlled incubators. All animals had *ad libitum* access to food and water. The piglets were weighed at the beginning and end of the experiment, and the feed intake was recorded daily to calculate the average daily gain (ADG), average daily feed intake (ADFI), and gain-to-feed ratio (G:F).

### 2.2 Diarrhea score

During the whole feeding trial period, the diarrhea scores of 1–3, where 1 indicates normal stools, 2 indicates semi-watery diarrhea, and 3 indicates watery diarrhea, were recorded twice a day (10:00 and 16:00) by counting the number of pigs with diarrhea per metabolism cage.

### 2.3 Slaughter procedure

Before the end day of the trial, all piglets fasted overnight, and they were slaughtered by administering an intravenous injection of sodium pentobarbital (50 mg/kg BW, Sigma) on the last day of the trial. Blood samples from the anterior vena cava were collected, and serum samples were isolated from the blood after centrifugation for 10 min at 3,000×g and 4°C. All samples were then held at −80°C for analysis.

### 2.4 pH analysis of the gastric, distal ileum, and colon contents

After the slaughter, gastric, distal ileum, and colon contents were collected. A pH meter (AB15 Basic, Thermo Fisher Scientific Inc., Waltham, MA) was placed in the digesta to measure the pH level.

### 2.5 Proteinase analysis

After the piglets were slaughtered, the gastric and distal ileum contents were collected, and the concentrations of chymotrypsin, pepsin, and trypsin were measured using assay kits (Nanjing Jiancheng Bioengineering Institute, Nanjing, China).

### 2.6 Biochemical analysis of serum

The serum was measured using commercially available porcine-specific kits. The levels of total protein (Diener and Scharrer, 1997; TP, Gen.2, 300Tests, cobas c, Integra Reagents, kits); albumin (ALB; BCG Gen.2, 300Tests, cobas c, Integra Reagents, kits); urea (UREAL; 500Tests, cobas c, Integra · Reagents, kits); high-density lipoprotein (HDL; Gen.3, 200Tests, cobas c, Integra · Reagents, kits); low-density lipoprotein (LDL; Gen.2, 175Tests, cobas c, Integra · Reagents, kits); immunoglobulin G (IgG; Gen.2, 150Tests, cobas c · Reagents, kits); immunoglobulin M (IgM; Gen.2, 150Tests, cobas c · Reagents, kits); Ca (Gen.2, 300Tests, cobas c, Integra); and serum osmotic-related ions (Na^+^, K^+^, and Cl^−^) were determined using assay kits (Nanjing Jiancheng Bioengineering Institute, Nanjing, China), following the manufacturer’s instructions. Furthermore, all the biochemical parameters were analyzed by atomic absorption spectrometry.

### 2.7 Real-time PCR assays

The total RNA was extracted from the ileum tissue, which was frozen in liquid nitrogen and fractured using TRIzol reagent (Invitrogen, United States), and added into DNase I (Invitrogen, United States; Laforenza, 2012; Thiagarajah et al., 2015). The reverse transcription program was set at 37°C for 15 min and 95°C for 5 s. Primers used for this experiment were designed using Primer 5.0 based on pig gene sequences (Table 2). Beta-actin was used as the housekeeping gene to normalize target gene mRNA levels. The PCR cycling conditions were set at 36 cycles at 94°C for 40 s, 60°C for 30 s, and 72°C for 35 s. The relative expression was calculated as the ratio of the target gene to the control gene using the formula 2^−∆∆Ct^, where ∆∆Ct refers to the Ct Target-Ct beta-actin treatment − Ct Target-Ct beta-actin control. The relative expression was normalized and expressed as a ratio to the expression in the control group (Kellenberger and Schild, 2015).

**Table 2:** Primers used for real-time quantitative PCR.

Accession no.	Gene	Primers (5′-3′)	Product length (bp)
NM_001164021.1	SLC5A1	F: AATGCGGCTGACATCTCTGT	95
		R: CCAACGGTCCCACGATTAGT	
NM_001012298.2	SLC26A6	F: AATGCGGCTGACATCTCTGT	92
		R: CCAACGGTCCCACGATTAGT	
XM_003123899.5	SLC12A2	F: ATGGGGCTGTCAGAAGCG	135
		R: ACATGGTAGTCTCGCCTCCT	
XM_021077062.1	SLC9A3	F: ATCGCCTGGTGTCAAGGATG	129
		R: GGTTGAGTTGGAGTCTTGCC	
XM_021101946.1	SLC26A3	F: GTGTACCCCTGGGCACTTG	126
		R: CCACAGCGTGAGGAGGAAG	
XM_003124280.3	beta-actin	F: CCTCGCCTGACTGTCAAAGA	147
		R: TGTTAGAACAATCTCGTTCCAAAGT	
NM_001272042.1	RPS6KB1	F: CTGCGGCATCCACGAAACT	189
		R: AGGGCCGTGATCTCCTTCTG	

SLC9A3, sodium/hydrogen exchanger 3; SLC12A2, sodium–potassium–chloride (Na–K–Cl) cotransporters; SLC26A3, Cl^−^/HCO_3_^−^ exchanger (DAR); SLC26A6, Cl^−^/HCO_3_^−^ exchanger (PAT1); SLC5A1, sodium/glucose cotransporter 1; and RPS6KB1, phosphorylated 40S ribosomal S6 kinases.

### 2.8 Western blotting analysis

The antibodies used for protein quantification in our study are as follows: beta-actin (60008-1, 1:5,000, Proteintech), RPS60KB1 (A16968, 1:1,000 ABclonal), SLC9A3 (AB2756978, 1:1,000 ABclonal), SLC12A2 (8351S, 1:2,000 CST), SLC26A3 (5642S, 1,1,000 CST), SLC26A6 (A10323, 1:1,000 ABclonal), and SLC5A1 (A13164, 1:1,000 ABclonal), along with the Horseradish Peroxidase (HRP)-conjugated secondary antibodies. We digitally quantified the resultant signals and normalized the data to beta-actin abundance. The data are expressed relative to the values in the piglets in the LP group.

### 2.9 16S rRNA sequencing and characterization of the microbiota

The sample collection, handling, and analytical methods are described in previous articles (Gao et al., 2020).

### 2.10 Statistical analysis

Student’s t-test was used to analyze the data using IBM SPSS 21.0 software and IIME software (Version 1.7.0) to ensure the homogeneity of variances.

## 3 Results

### 3.1 Effect of different protein level diets on growth performance

**Figure 1:**
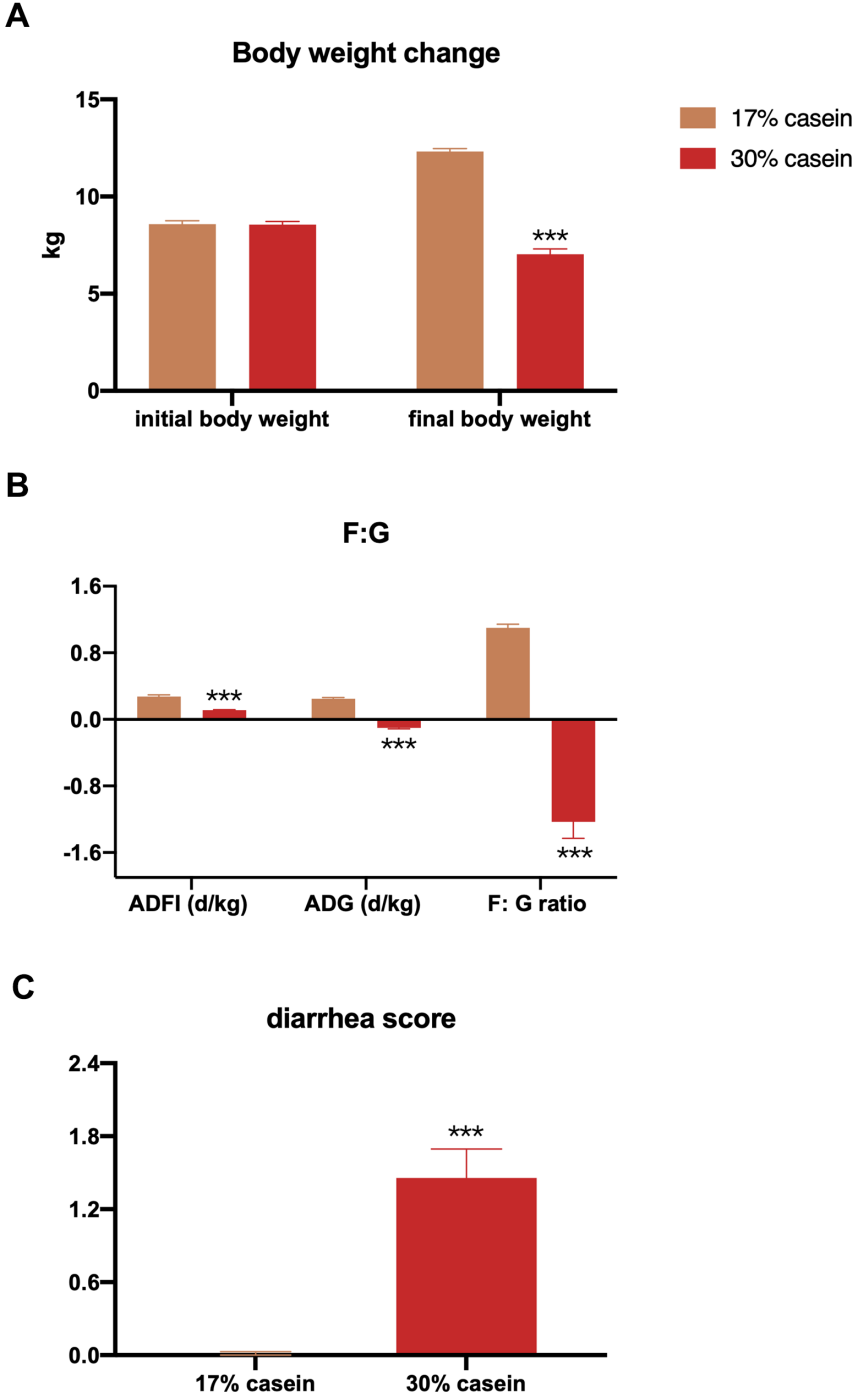
Effect of different protein level diets on the growth performance of post-weaning piglets. **(A)** Body weight change. **(B)** Average daily gain of body weight (ADG), average daily feed intake (ADFI), and the ratio of ADFI/ADG (F:G). Values are expressed as mean ± SEM, *n* = 7. ^*^p < 0.05, ^**^p < 0.01, ^***^p < 0.001, and ns, *p* > 0.05.

The results indicated that a high-protein feed induced persistent diarrhea in piglets throughout the study, whereas no diarrhea was observed in control piglets (*p* < 0.05) (Figure 1C). In addition, the body weight change (*p* < 0.05), average daily gain (ADG; *p* < 0.05), average daily feed intake (ADFI; *p* < 0.05), and feed efficiency (F:G ratio; *p* < 0.05) were reduced in the HP group compared to the control piglets (Figures 1A,B).

### 3.2 Effect of different protein level diets on the pH levels of gastric, ileum, and colon contents

**Figure 2:**
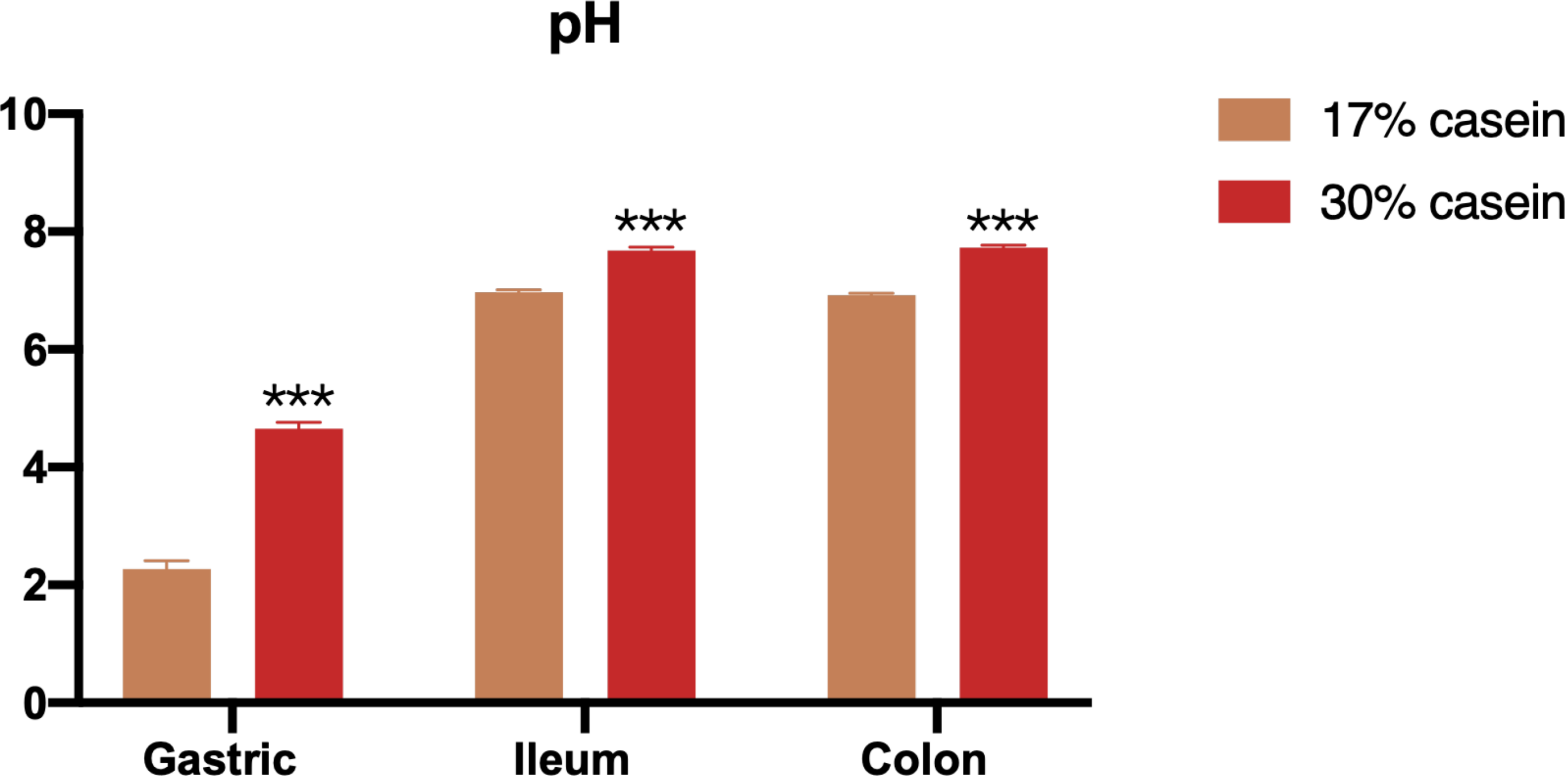
Effect of different protein level diets on the gastric, ileum and colon pH of post-weaning piglets. Values are expressed as mean ± SEM, *n* = 7. ^*^p < 0.05, ^**^p < 0.01, ^***^p < 0.001, and ns, *p* > 0.05.

According to our study, the pH levels of the gastric, distal ileum, and colon contents were significantly higher in piglets in the HP group than those in the LP group (Figure 2).

### 3.3 Effect of different protein level diets on protease activity

**Figure 3:**
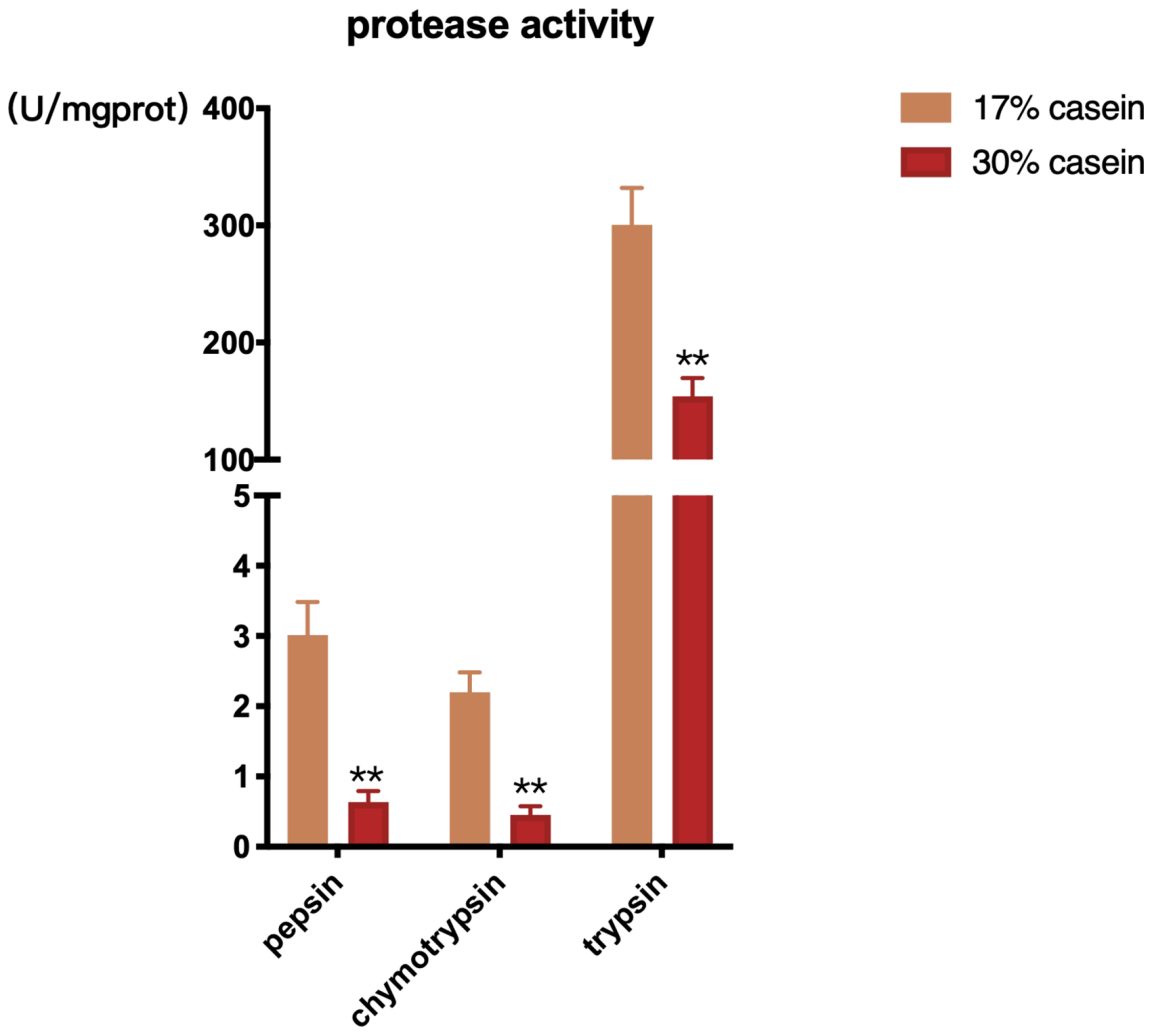
Effect of different protein level diets on protease activity of post-weaning piglets. Values are expressed as mean ± SEM, *n* = 7. ^*^p < 0.05, ^**^p < 0.01, ^***^p < 0.001, and ns, *p* > 0.05.

Feed with high-casein concentration was found to inhibit the activity of chymotrypsin, pepsin, and trypsin in the distal ileum of piglets in the HP group compared to those in the control group (Figure 3).

### 3.4 Effect of different protein level diets on serum biochemistry

**Figure 4:**
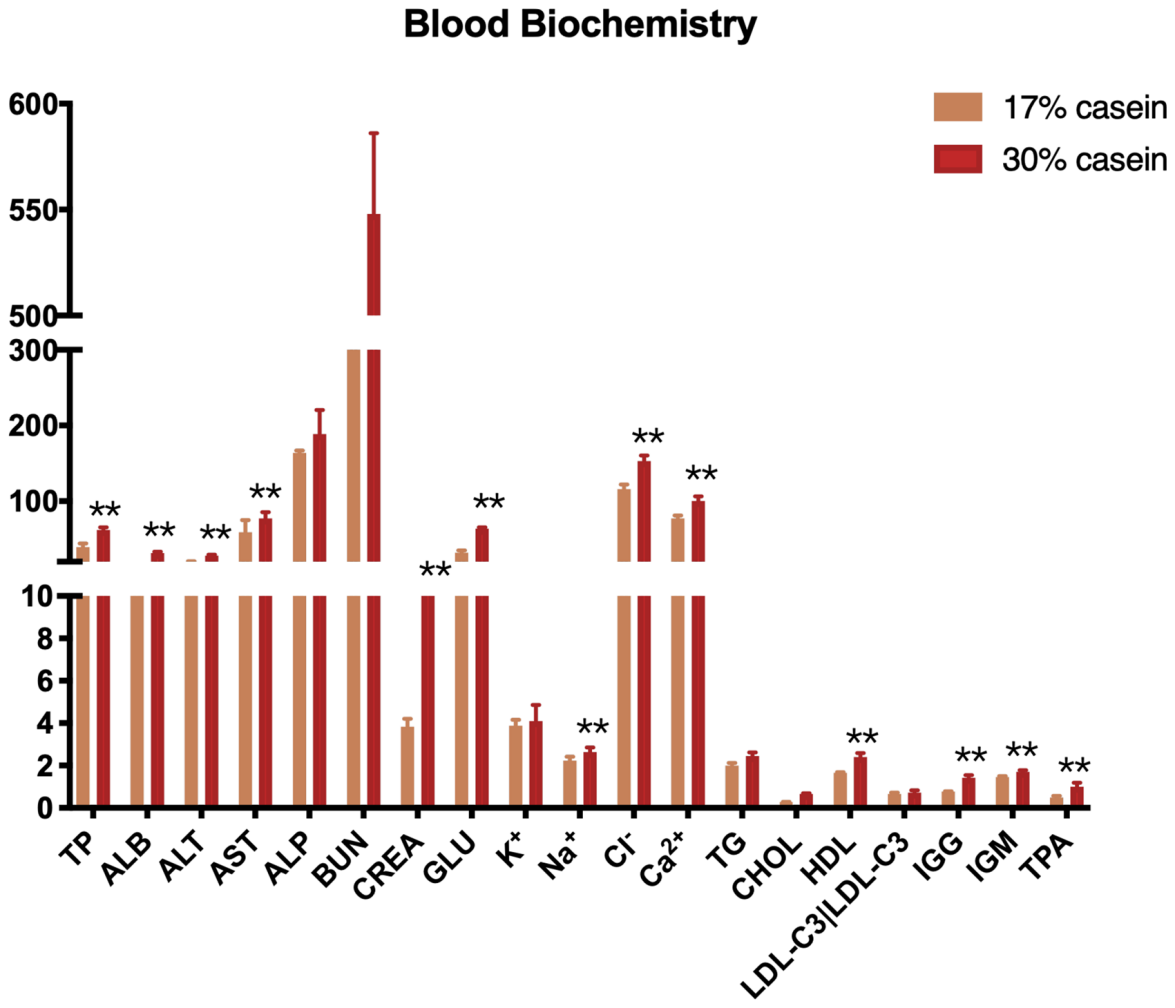
Effect of different protein level diets on the serum biochemistry of post-weaning piglets. Values are expressed as mean ± SEM, *n* = 7. ^*^p < 0.05, ^**^p < 0.01, ^***^p < 0.001, and ns, *p* > 0.05.

In our study, inflammation-related indices, including total protein (TP), albumin (ALB), alanine aminotransferase (ALT), glutamic oxaloacetic transaminase (AST), creatinine (CREA), glucose (Glu), high-density lipoprotein (HDL), immunoglobulin G (IgG), immunoglobulin M (IgM), and tissue plasminogen activator (TPA), were significantly higher in piglets in the HP group than those in the LP group. However, LDL levels did not differ between piglets in the HP and those in the LP groups. Osmotic/ion-exchange-related indices, including TP, Na^+^, Cl^−^, and Ca^2+^, were significantly higher in piglets in the HP group than those in the LP group, whereas K^+^ did not differ in both groups (Figure 4).

### 3.5 Effect of different protein level diets on the relative gene expression of ion transporter carriers

**Figure 5:**
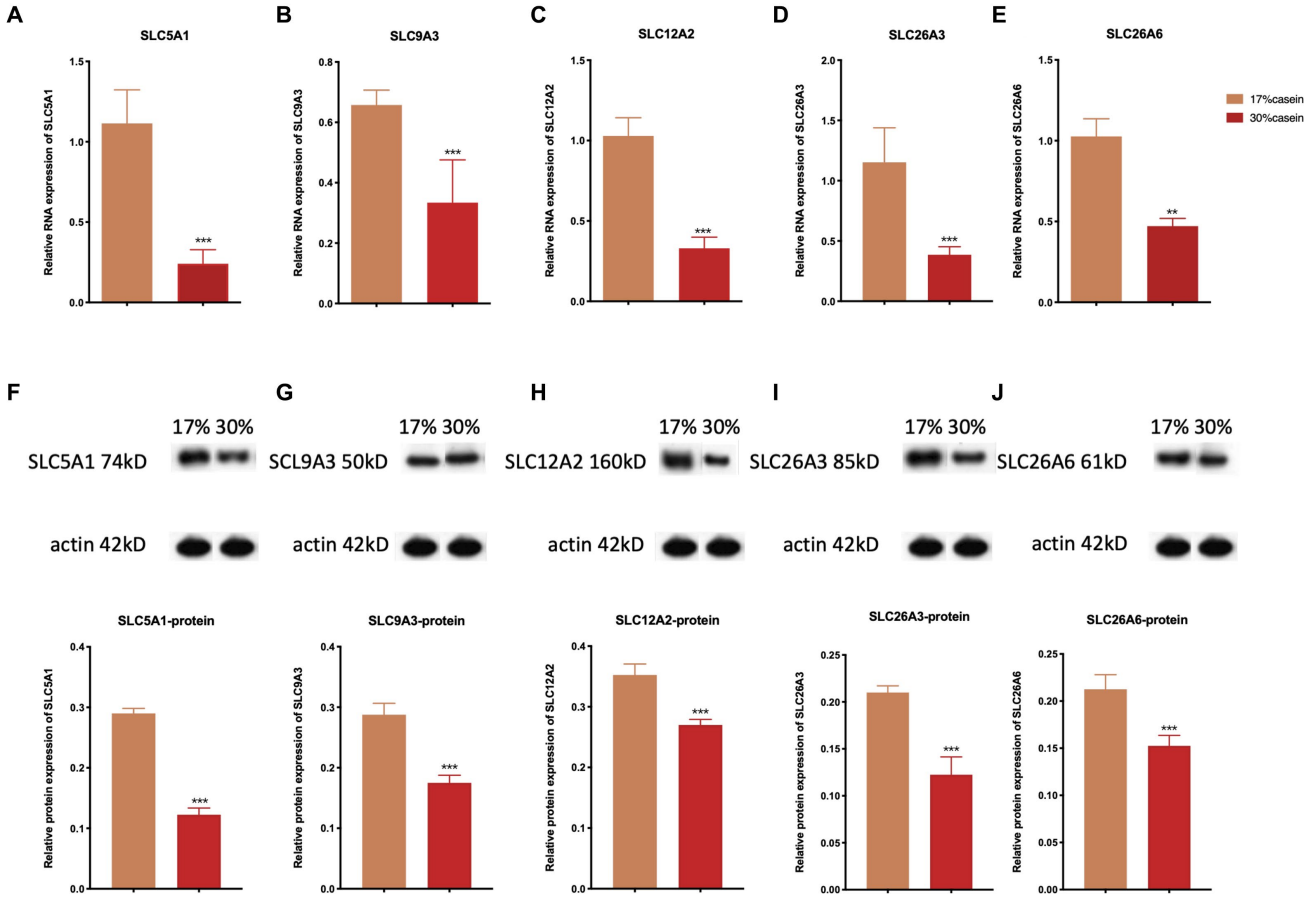
Effect of different protein level diets on the relative gene expression of ion transporter carriers of post-weaning piglets. The relative expression of **(A,F)** SLC5A1, **(B,G)** SLC9A3, **(C,H)** SLC12A2, **(D,I)** of SLC26A3, and **(E,J)** SLC26A6. Values are expressed as mean ± SEM, *n* = 7. ^*^p < 0.05, ^**^p < 0.01, ^***^p < 0.001, and ns, *p* > 0.05.

The results indicated that the relative gene expression and the protein expression of SLC12A2, SLC26A3, SLC9A3, SLC26A6, and SLC5A1 were significantly lower in the ileum tissue of piglets in the HP group than of those in the LP group (Figure 5).

### 3.6 Effect of different protein level diets on the gut microbial diversity and abundance

**Figure 6:**
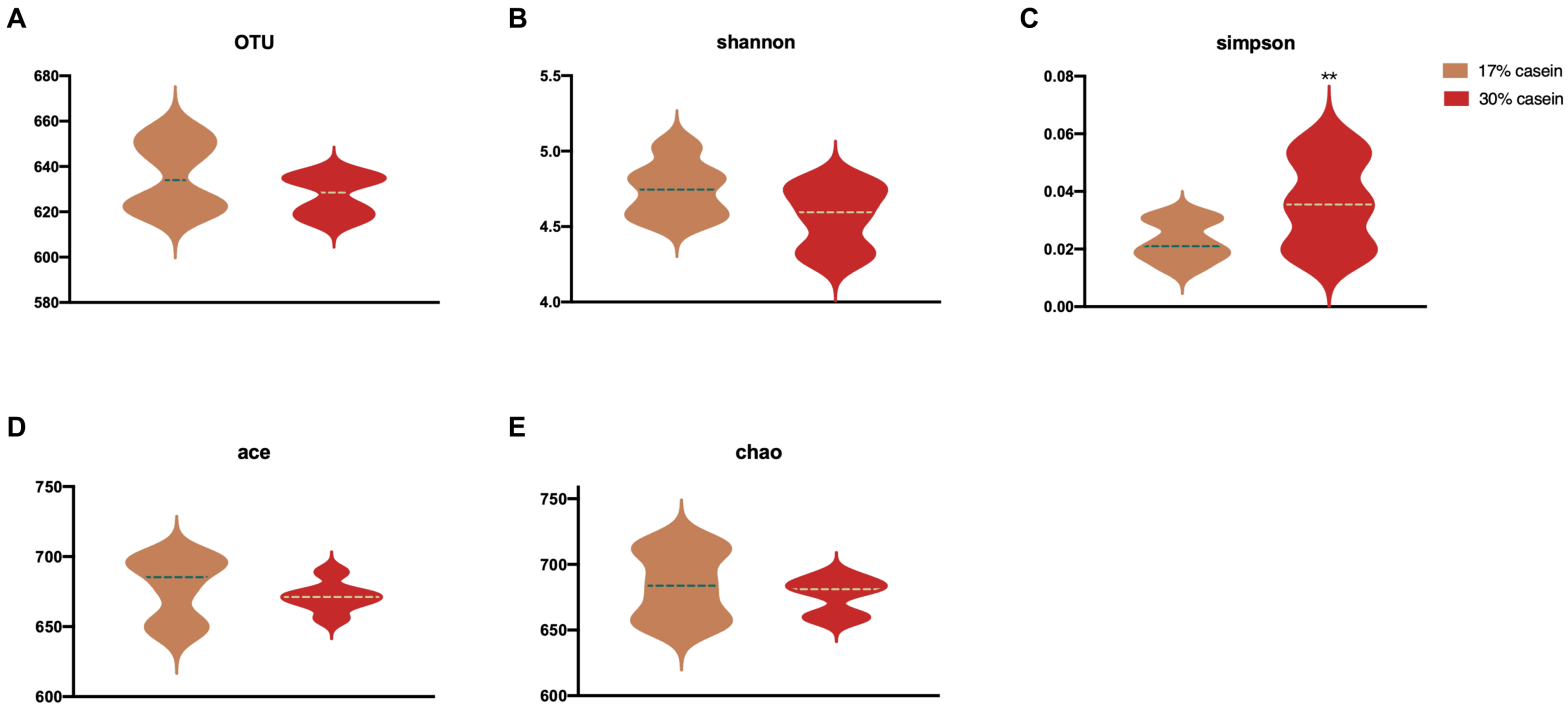
Effect of different protein level diets on the gut microbial diversity of post-weaning piglets. **(A)** OTUs. **(B)** Shannon H index. **(C)** Simpson. **(D)** ACE. **(E)** Chao1 index. Values are expressed as mean ± SEM, *n* = 7. ^*^p < 0.05, ^**^p < 0.01, ^***^p < 0.001, and ns, *p* > 0.05.

Highly variable V3 and V4 regions of the 16S rRNA gene were sequenced based on fecal samples, with an average of 79,920 ± 5,250 sequences detected per sample. Sequences with ≥97% similarity within the reading frame were selected, and an average of 67,899 ± 100 operational taxonomic units (OTUs) was obtained. α-Diversity among microbial communities was assessed using Shannon (4.47 ± 0.07 and 4.17 ± 0.18, *p* = 0.18), Simpson (0.02 and 0.05 ± 0.01, *p* = 0.03), ACE (0.02 and 0.05 ± 0.01, *p* = 0.65), and Chao1 (684.2 ± 10.71 and 676.81 ± 4.97, *p* = 0.55) indices. Diets with high-casein concentration (i.e., the HP diet) significantly reduced microbiota diversity than those with low-casein concentration (i.e., the LP diet; Figure 6).

**Figure 7:**
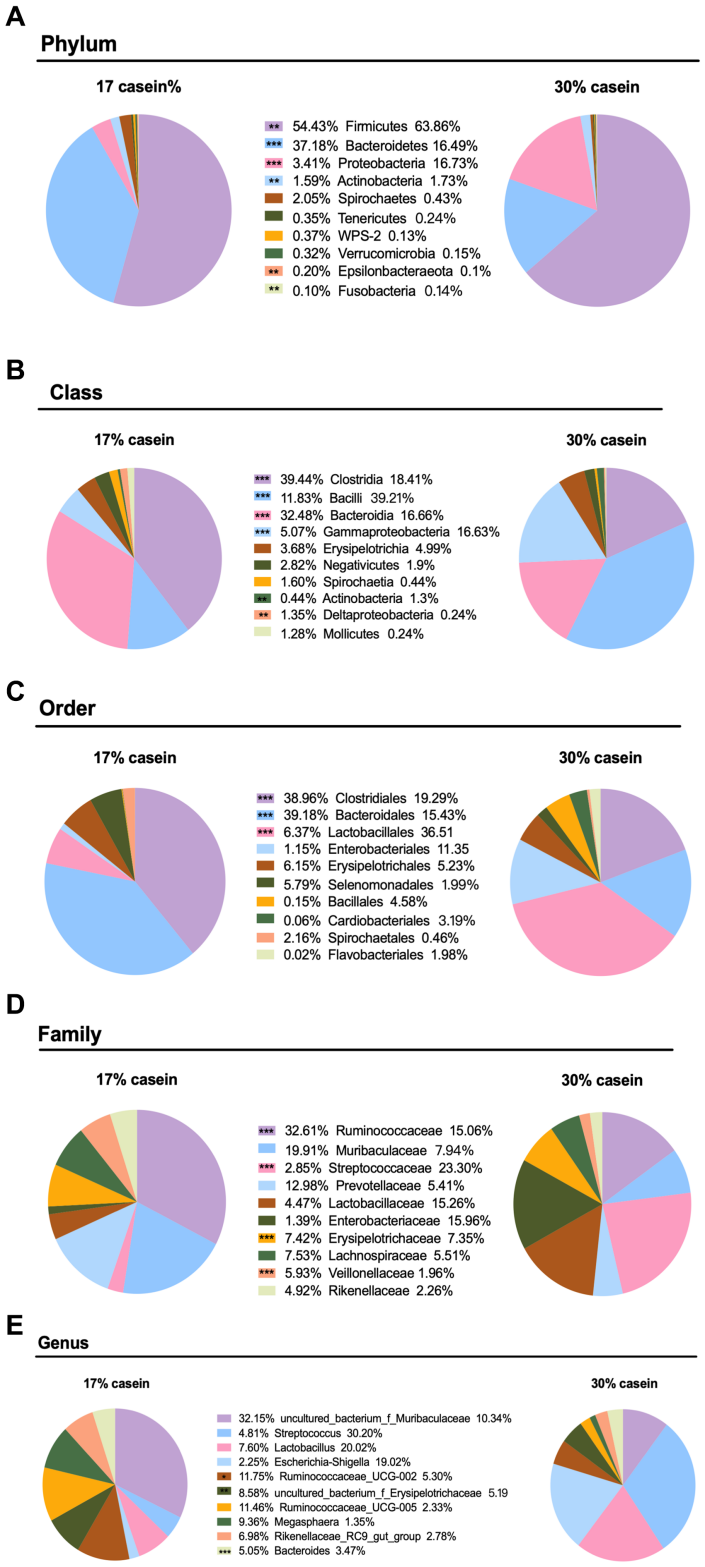
Effect of different protein level diets on the gut microbial abundance of post-weaning piglets. The composition of microbiota at the **(A)** phylum level, **(B)** class level, **(C)** order level, **(D)** family level, and **(E)** genus level. Values are expressed as mean ± SEM, *n* = 7. ^*^p < 0.05, ^**^p < 0.01, ^***^p < 0.001, and ns, *p* > 0.05.

Increasing the dietary protein level significantly affected the microbial composition of weaned piglets at several levels. At the phylum level, 30% dietary casein significantly decreased the relative abundance of *Bacteroidetes* (*p* < 0.05), *Spirochaetes* (*p* < 0.05), *Cyanobacteria* (*p* < 0.05), and *Elusimicrobia* (*p* < 0.05), but it significantly increased the relative abundance of *Firmicutes* (*p* < 0.05), *Proteobacteria* (*p* < 0.05), and *Actinobacteria* (*p* < 0.05). At the class level, high-casein-concentration diets significantly decreased the fecal abundance of *Clostridia* (*p* < 0.05), *Proteobacteria* (*p* < 0.05), *Actinobacteria* (*p* < 0.05), *Bacteroidia* (*p* < 0.05), *Deltaproteobacteria* (*p* < 0.05), and others (*p* < 0.05), whereas it significantly increased the relative abundance of *Bacilli* (*p* < 0.05), *Gammaproteobacteria* (*p* < 0.05), and *Actinobacteria* (*p* < 0.05). At the order level, LP piglets fed with 30% casein had a lower relative abundance of *Clostridiales* (*p* < 0.05) and *Bacteroidales* (*p* < 0.05) and a higher relative abundance of *Lactobacillales* (*p* < 0.05). At the genus level, feeding 30% casein to weaned piglets significantly reduced the relative abundance of *Ruminococcus* (Figures 7A–E).

### 3.7 Effect of different protein level diets on the relative expression of the mTOR signal pathway

**Figure 8:**
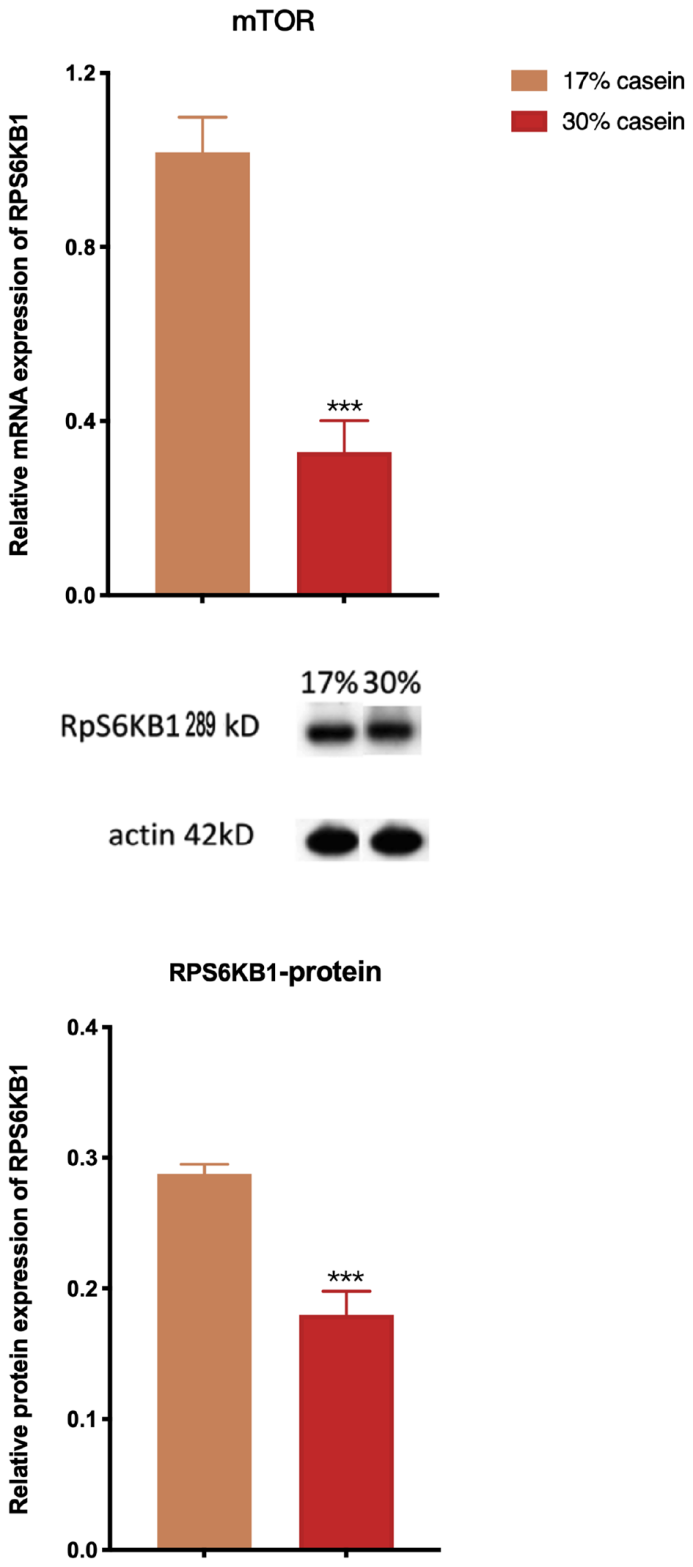
Effect of different protein level diets on the relative expression of the mTOR pathway signal of post-weaning piglets. Values are expressed as mean ± SEM, *n* = 7. ^*^p < 0.05, ^**^p < 0.01, ^***^p < 0.001, and ns, *p* > 0.05.

The results of the real-time PCR assays and western blotting analysis showed that a diet with high-casein concentration decreased the relative gene expression and protein expression of the mTOR (*p* < 0.05). This finding indicates that a diet with high-casein concentration inhibited the mTOR signal pathway compared to a diet with low-casein concentration (Figure 8).

## 4 Discussion

Many animal models have been used to better understand the impact of nutritional restrictions on the health of humans, yielding significant results in several intestinal diseases (Anbazhagan et al., 2018). Weaning of piglets is characterized by their immature immune system and incompletely developed digestive system, making nutritional composition a key factor to be considered during this period (Canessa et al., 1993). Protein is a fundamental part of healthy nutritional composition in animals and humans, and the concentration and sources of dietary protein are reported to be closely associated with post-weaning diarrhea in piglets and other animals (Bannon et al., 2003; Bamias et al., 2017). In this study, we investigated two diets with different protein concentrations to model the common occurrence of post-weaning diarrhea in piglets. A diet containing 30% protein significantly decreased the growth performance and caused severe diarrhea.

Proteins are found in high concentrations in the small intestines and feces. The degradation products of dietary proteins, such as free amino acids and other organic nitrogen-containing compounds, are detected in the distal colon contents and are available for fermentation by the intestinal microbiota at millimolar concentrations (Mizoguchi, 2012). After initial digestion, residual dietary proteins and enzymes in the small intestine reach the colon, where they are fermented by the intestinal microbiota. The primary fermentation products of amino acids include ammonia and branched-chain fatty acids (Thanikachalam and Khan, 2019). Previous studies have shown that low pH levels can decrease the products of amino acid fermentation by colonic bacteria, specifically amines (DiMaggio et al., 2017). The pH level of the gastric chamber ranges from 2.0 to 3.5, the pH level of the distal ileum ranges from 5.0 to 7.0, and the pH level of the colon ranges from 5.5 to 7.0 (Caffarelli et al., 2018). The acidic environment of the gastric chamber aids in the digestion of dietary protein in mammals, promoting the secretion of pepsinogen and its activation to pepsins in the stomach (Bailey et al., 2005). At higher pH levels of 6.5 to 8.0, pepsin exhibits no activity (Heo et al., 2013). The pH in the ileum and colon has an important selective effect on the microbial population, with a normal pH level inhibiting the prevalence of *Bacteroides* spp. and promoting butyrate-producing gram-positive bacteria (Gilbert et al., 2018). This phenomenon is largely associated with a low concentration of short-chain fatty acids (Heo et al., 2015). The pH level is also closely associated with the balance of osmotic pressure and permeability of the gastrointestinal membrane (Karlund et al., 2019). Disordered gastrointestinal pH disrupts the secretion of ion exchangers such as SLC12A2, SLC26A3, SLC9A3, SLC26A6, and SLC5A1 in the ileum of piglets, thereby causing severe diarrhea (Schutz, 2011). Consistent with previous studies, in our study, the HP group had a significantly increased pH value in the stomach, ileum, and colon. This phenomenon is likely associated with the extent of degradation of dietary proteins because undigested protein accumulates in the gastrointestinal tract and disrupts the balance of osmotic pressure. This disruption can result in disordered ion exchange and increased cell permeability, thereby increasing the concentration of ammonia and inducing post-weaning diarrhea.

Digestive enzymes serve as important regulators of the digestive system (Yao et al., 2016) and are involved in the activation of inflammatory pathways as well as the disruption of the immune system (Singh and Garg, 2016; Ma et al., 2017). For example, the premature activation of trypsin can cause acute pancreatitis (Shortt et al., 2018). The digestion of proteins in the gastrointestinal tract mainly depends on the activity of proteases, including trypsin and chymotrypsin (Hu and Zhang, 2018; Kobayashi, 2018). Pepsinogens are secreted by the stomach, and for optimal pepsinogen activity, the pH level should be 1.8–3.5. Moreover, pepsinogens lose their optimal activity in an alkaline environment (Schubert, 2014; Espinoza et al., 2018). The pancreas is the major source of proteases in the digestive system that facilitate the digestion of dietary proteins. Trypsinogen and chymotrypsinogen are two important products of the pancreas (Armstrong, 2004). While trypsinogen is reportedly involved in regulating other digestive enzymes, the activation of trypsin is pH-dependent, with trypsin maintaining its optimal activity in an environment with pH levels of 7.5–8.5 (Kaunitz and Tanaka, 1996). Several studies have indicated that trypsin and chymotrypsin may be involved in the activation of the immune system in humans (Johnston et al., 2018). A dysregulation in the protease balance has been reported to be closely associated with gastrointestinal diseases such as diarrhea (Petersen, 2018). The major function of those proteases is protein digestion. In our study, the HP group exhibited significantly reduced concentrations of pepsins, trypsin, and chymotrypsin, resulting in the accumulation of undigested proteins. These proteins induced toxic products that were harmful to the gastrointestinal tracts and immune systems of the piglets.

Previous studies have shown that fluid absorption is dependent on the concentrations of Na^+^ across the epithelium, combined with the absorption of Cl^−^ or HCO3^–^ (Amidon et al., 2015). Basolateral Na^+^/K^+^-ATPase can also act as an energy provider during the ion exchange process (Esseku and Adeyeye, 2011). In the small intestine, fluid absorption is driven by sodium/hydrogen exchanger 3 (SLC9A3), sodium/glucose cotransporter 1 (SLC5A1), and the Cl^−^/HCO3^−^ exchangers SLC26A3 and SLC26A6 (Gregory, 1996; Tan et al., 2014). Fluid absorption in the colon depends on the activity of SLC9A3 combined with SLC26A and SLC26A6 (Stock and Pedersen, 2017). The substrate-specific transporters SLC5A1 can also increase the action of Na^+^ across the apical membrane. Many reports have indicated that the movement of Ca^2+^ suppresses the activity of SLC5A1 and SLC9A3. Therefore, intestinal fluid secretion is facilitated by transepithelial Cl^−^ secretion via the Na^+^/K^+^/2Cl^−^ cotransporter (SLC12A2); moreover, the Na^+^ concentration gradient generated by Na^+^/K^+^-ATPases can act as energy providers (Janecke et al., 2016). Previous studies have demonstrated that SLC12A2 may be an independent regulator of the rate of epithelial secretion. The ion exchange process undertaken by ion-transporting proteins plays an important role in several metabolic functions of the host, such as assisting digestive enzymes and secretory immune cells. Moreover, the fluid absorption process is closely associated with intestinal diseases such as diarrhea and inflammatory bowel diseases (Dawson and Markovich, 2005; Cseko et al., 2019). The inhibition of epithelial Cl^−^ secretion, or mutation of SLC12A2, can strengthen the incidence of diarrhea (Janecke et al., 2016). Several studies have shown that modulating the expression of SLC26A3 and SLC26A6 can cause severe high-chloride diarrhea (Alper and Sharma, 2013). SLC9A3 expression is also inhibited by the expression of IL-10, inducing diarrhea (Whitcomb and Lowe, 2007). In our study, the relative mRNA and protein expression of SLC12A2, SLC26A3, SLC9A3, SLC26A6, and SLC5A1 were inhibited in piglets with a high-protein diet. The concentrations of Na^+^ and Cl^−^ were also higher in piglets in the HP group than in the LP group. The imbalance of ion transporters induced by the accumulation of undigested proteins may disrupt intestinal fluid absorption, misregulate intestinal osmotic pressure, and inhibit the transport of digestive enzymes, which results in the inhibition of protein digestion and finally causes diarrhea.

The gut flora supports a healthy intestinal environment by suppressing pathogen components, promoting a beneficial immune system, and encouraging the development of balanced microbiome on the host (Wang and Ji, 2019). We examined the microorganisms of two diet groups and found that the composition and relative abundance of intestinal tract microorganisms of piglets were significantly affected by the protein concentration in the diet. Most human gut microorganisms belong to the *Firmicutes*, *Bacteroidetes*, and *Actinobacteria* phyla, which play a key role in nutrient absorption. *Actinomycetes* are Gram-positive filamentous bacteria, which are considered potential probiotics and are important for the maintenance of intestinal homeostasis (Minty et al., 2010). *Bifidobacterium*, belonging to the phylum *Actinomycetota*, and *Lactococcus*, belonging to the phylum *Bacillota*, are the most abundant microbial groups in the intestinal tract, especially in the neonatal gastrointestinal tract. They contribute to the development of host intestinal physiology, such as immune system maturation, digestion, bacterial colonization, and pathogen suppression. In our study, a diet with 30% casein decreased the abundance of *Firmicutes*, *Bacteroidetes*, and *Actinobacteria* phylum in the intestinal tracts of the piglets, consistent with the findings of previous studies. Dozens of studies have shown that *Clostridium* spp. is closely associated with colorectal cancer and *Escherichia coli* contributes to diarrhea in humans and pigs (Gao et al., 2019). Our results are consistent with reports that *E. coli* causes diarrhea in piglets. The low levels of protein digestion may have increased the pH value in the gastrointestinal tract of piglets, creating a favorable environment for the proliferation of *E. coli* and other potential pathogens, thereby inducing diarrhea.

The mTOR signaling integrates nutrient signals and metabolism in response to environmental factors such as nutrients and growth factors (Mossmann et al., 2018). It is essential for protein production and cellular growth signals (Kim and Guan, 2019). Amino acids, specifically leucine and arginine, stimulate mTOR signaling and are associated with the regulation of animal physiology (Weichhart, 2018). The mammalian intestinal epithelium, composed of enterocytes, goblet cells, and enteroendocrine cells, plays a key role in establishing regenerative capacity after injury. mTORC1 signaling is also involved in intestinal regeneration and the host immune system (Wei et al., 2019). We observed that a high-protein diet induced inflammation and significantly increased the expression of the immune response factors IgG and IgM, suggesting that a high-protein diet leads to intestinal inflammation and injury in piglets. This may be due to the inhibition of the mTOR signaling pathway and a reduction in the capacity of the intestinal epithelium to regenerate. Alternatively, the elevated intestinal pH and inhibited protease activity observed in the high-protein diet group, along with the disrupted ion exchange process, inhibited protein digestion. This inhibition of protein digestion resulted in a shortage of amino acids, such as leucine and arginine, leading to the downregulation of the mTOR signaling pathway. The inhibition of the mTOR signaling pathway in turn increased the incidence and severity of post-weaning diarrhea in piglets.

## 5 Conclusion

This study investigated the effect of dietary protein levels on the health and development of post-weaning diarrhea in piglets. The results indicated that a diet with 30% protein decreased growth performance and induced severe diarrhea. This result may be attributed to the accumulation of undigested proteins in the gastric chamber and intestines, disrupting the gastric, distal, and colon pH levels. In addition, the expression of chymotrypsin, pepsin, and trypsin as well as ion exchangers was inhibited. The microorganisms of the two diet groups were significantly affected by the protein concentration of the diet. These factors may cause a dysfunctional immune system and digestive system. Importantly, the high-protein diet inhibited the mTOR signaling pathway, which increased the incidence and severity of post-weaning diarrhea in piglets.

## Data availability statement

The raw data supporting the conclusions of this article will be made available by the authors, without undue reservation.

## Ethics statement

The studies involving humans were approved by with the ethical approval code ISA2017030523, the Chinese Academy of 487 Sciences’ Animal Care and Use Committee accepted the experimental protocol. The studies were conducted in accordance with the local legislation and institutional requirements. Written informed consent for participation in this study was provided by the participants’ legal guardians/next of kin. The animal studies were approved by with the ethical approval code ISA2017030523, the Chinese Academy of 487 Sciences’ Animal Care and Use Committee accepted the experimental protocol. The studies were conducted in accordance with the local legislation and institutional requirements. Written informed consent was obtained from the owners for the participation of their animals in this study.

## Author contributions

JG: Writing – original draft. LM: Writing – review & editing. YY: Writing – review & editing. YC: Funding acquisition, Supervision, Writing – review & editing. TL: Funding acquisition, Methodology, Project administration, Supervision, Writing – review & editing.
